# Significant changes in macrophage and CD8 T cell densities in primary prostate tumors 2 weeks after SBRT

**DOI:** 10.1038/s41391-022-00498-6

**Published:** 2022-01-20

**Authors:** Nathanael Kane, Tahmineh Romero, Silvia Diaz-Perez, Matthew B. Rettig, Michael L. Steinberg, Amar U. Kishan, Dorthe Schaue, Robert E. Reiter, Beatrice S. Knudsen, Nicholas G. Nickols

**Affiliations:** 1 Department of Radiation Oncology, David Geffen School of Medicine at UCLA, Los Angeles, CA USA; 2 Statistic Core, David Geffen School of Medicine at UCLA, Los Angeles, CA USA; 3 Department of Urology, David Geffen School of Medicine at UCLA, Los Angeles, CA USA; 4 Department of Pathology, University of Utah, Salt Lake City, UT USA; 5 Radiation Oncology Service, VA Greater Los Angeles Healthcare System, Los Angeles, CA USA

**Keywords:** Prostate cancer, Cancer therapy

## Abstract

**Background:**

Radiotherapy impacts the local immune response to cancers. Prostate Stereotactic Body Radiotherapy (SBRT) is a highly focused method to deliver radiotherapy often used to treat prostate cancer. This is the first direct comparison of immune cells within prostate cancers before and after SBRT in patients.

**Methods:**

Prostate cancers before and 2 weeks after SBRT are interrogated by multiplex immune fluorescence targeting various T cells and macrophages markers and analyzed by cell and pixel density, as part of a clinical trial of SBRT neoadjuvant to radical prostatectomy.

**Results:**

Two weeks after SBRT, CD68, and CD163 macrophages are significantly increased while CD8 T cells are decreased. SBRT markedly alters the immune environment within prostate cancers.

Neoadjuvant trials of radiation prior to surgery offer the unique opportunity to study irradiated tissues in situ. We previously reported a Phase 1 trial of stereotactic body radiation therapy (SBRT), three fractions of 8 Gy delivered over 1 week, 2 weeks prior to radical prostatectomy (RP) for high-risk prostate cancer [1]. Pretreatment biopsies and surgical specimens from this trial were used to determine radiation-induced changes in T cell and macrophage subsets by multiplex immune-fluorescence (mIF).

The 6 out of 11 cases were chosen due to their availability of biopsy and RP cores for use. Biopsy cores were selected for analysis based on tumor content greater than 70%. For each case, the tissue block with the dominant tumor nodule in the radical prostatectomy was identified by hematoxylin and eosin staining prior to mIF. Parallel slides were stained with antibodies against T cells [CD3 (clone 2Gv6 rabbit mAb, Ventana cat. 790-4341), CD4 (clone SP35 rabbit mAb, Ventana cat 790-4423), CD8 (clone SP57 rabbit mAb, Ventana cat. 790-4460), FoxP3 (clone SP97 rabbit mAb, Spring Bioscience cat. M3970)] or macrophages [CD68 (clone KP-1 mouse mAb, Ventana cat. 790-2931), CD163 (clone MRQ26 mouse mAb, Ventana cat. 760-4437), CD11b (clone EPR1344 rabbit mAb, Abcam cat. Ab133357)]. CD3 and CK8/18 (clone 5D3 mouse mAb, Abcam cat. Ab17139) antibodies were included in both panels. FFPE tissue sections were stained on the Ventana Discovery Ultra autostainer, and analyzed using Tissuegnostics Strataquest software (v. 6.0.1.211). A representative image is shown in Fig. 1. RP regions went through additional manual quality control with the guidance of a pathologist to exclude normal glands that were intermixed with tumor glands. Image analysis included optimization of IF thresholding and nuclear segmentation. A CK 8/18 defined mask in tumor regions was expanded by 20 micron to generate a ring of ~1–2 full cell lengths around tumor glands and to enumerate T cell and macrophage pixel numbers, as described previously [2].

**Fig. 1:**
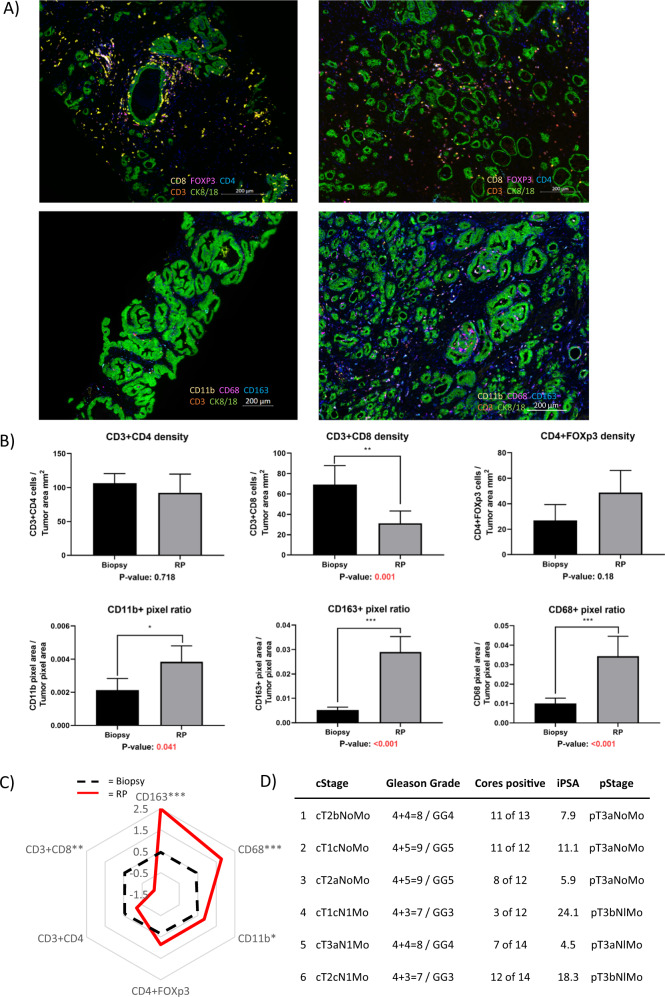
Changes in T cell and macrophage densities before and after prostate SBRT. **A** Representative multiplex IF images of a panel of T cell markers in pre-SBRT biopsies (top left) and post- SBRT RP specimens (top right) along with a panel of macrophage cell markers in biopsies (bottom left) and RP (bottom right). Images were taken using ZEN 3.1 imaging software. Sequential slides were cut at 4 µm prior to staining. The T cell panel included immune-fluorescent (IF) antibody-fluorophore pairs CD4-DCC, CD8-Rhod6G, CD3-Red610, FoxP3-Cy5, cytokeratin 8/18-FAM and DAPI. The macrophage panel included CD163-DCC, CD11b-Rhod6G, CD3-Red610, CD68-Cy5, cytokeratin 8/18-FAM and DAPI. **B** Bar graphs of T cells (top row) and macrophage cells (bottom row) within tumor regions. Means are shown with SEM error bars. T cells are counts of cells per unit area. Macrophages are positive pixels per unit area. **C** Polar graph using mean log2-fold differences between RP samples and biopsy samples log2(mean of RP/mean of biopsy). *p < 0.05, **p < 0.01, ***p < 0.001. **D** Clinical and pathologic features of cases analyzed.

We included each variable (T cell or macrophage densities or percentages of positive tumor region in pixels) as the dependent variable of a generalized linear mixed model (GLMM) for repeated measures in which RP vs Biopsy (RP y/n) was the independent covariate under a compound symmetry covariance structure matrix. All tests were two sided with *p* < 0.05 considered statistically significant. We used R (R version 3.6.1 (2019-07-05) and LME4 package and SAS (9.4, SAS Institute Inc., Cary, NC, USA) to analyze the data.

CD8 T cell (CD3 + CD8+) densities within tumor regions after SBRT were decreased 2.22-fold (*p* = 0.001) relative to densities before SBRT, while CD4 T cell (CD3 + CD4+) densities, and Treg cell (CD4 + FOXp3) densities remained largely unchanged. The most striking change was the increase in CD11b + myeloid cells, particularly the CD68 + macrophages, and CD163+ macrophage subsets that reached 1.85-fold, 3.43-fold, and 5.61-fold change in densities after SBRT, respectively (Fig. 1).

Altogether, the immune environment within prostate tumors is significantly altered 2 weeks after SBRT. This is the first report to quantify infiltrating immune cells in situ within prostate tumors to determine local effects of SBRT in patients. This is in line with our previous flow cytometric investigation showing an equivalent shift in infiltrating immune cells post SBRT towards myeloid lineage predominance although tumor and non-tumor regions could not be distinguished at that time [3]. By spatially resolving tumor and non-tumor glands in situ, we demonstrate significant increases in macrophage densities and moderate reductions in CD8 T cell densities 2 weeks post SBRT. In a syngeneic murine model, we previously reported increases in myeloid cells 2 days after high dose per fraction radiation, followed by a transient increase of functionally active CD8 lymphocytes [4]. The changes we report here in macrophage and CD8 T cell densities 2 weeks post SBRT in patients may also be time dependent, but our analysis was limited to a single time point given the design of the clinical trial. Similar digital spatial profiling of prostate tissue before and after 2 weeks of 10 Gy delivered by HDR brachytherapy also indicated such an increase in macrophage subpopulations in the post-radiation phase [5]. The fact that this was accompanied by a strong tumor inflammatory signature points at an integrated, myeloid-centric tissue response that radiation damage is known to initiate. To reveal underlying cytokines and mechanisms that are responsible for observed changes in the immune cell infiltration, transcriptomic analyses on prostate cancer tissue post SBRT are warranted. Ultimately, the questions will be whether or not these myeloid subsets are hindering the anti-tumor immune response and if so, how they can be targeted.
